# Rapid 3D absolute B_1_^+^ mapping using a sandwiched train presaturated TurboFLASH sequence at 7 T for the brain and heart

**DOI:** 10.1002/mrm.29497

**Published:** 2022-11-06

**Authors:** James L. Kent, Iulius Dragonu, Ladislav Valkovič, Aaron T. Hess

**Affiliations:** ^1^ Wellcome Centre for Integrative Neuroimaging, FMRIB, Nuffield Department of Clinical Neurosciences University of Oxford Oxford UK; ^2^ Siemens Healthcare Limited Frimley Surrey UK; ^3^ Oxford Centre for Clinical Magnetic Resonance Research (OCMR) University of Oxford Oxford UK; ^4^ Department of Imaging Methods Institute of Measurement Science, Slovak Academy of Sciences Bratislava Slovakia

**Keywords:** B_1_^+^ mapping, parallel transmit, satTFL, sandwich, ultrahigh field MRI

## Abstract

**Purpose:**

To shorten the acquisition time of magnetization‐prepared absolute transmit field (B_1_^+^) mapping known as presaturation TurboFLASH, or satTFL, to enable single breath‐hold whole‐heart 3D B_1_^+^ mapping.

**Methods:**

SatTFL is modified to remove the delay between the reference and prepared images (typically 5 T_1_), with matching transmit configurations for excitation and preparation RF pulses. The new method, called Sandwich, is evaluated as a 3D sequence, measuring whole‐brain and gated whole‐heart B_1_^+^ maps in a single breath‐hold. We evaluate the sensitivity to B_1_^+^ and T_1_ using numerical Bloch, extended phase graph, and Monte Carlo simulations. Phantom and in vivo images were acquired in both the brain and heart using an 8‐channel transmit 7 Tesla MRI system to support the simulations. A segmented satTFL with a short readout train was used as a reference.

**Results:**

The method significantly reduces acquisition times of 3D measurements from 360 s to 20 s, in the brain, while simultaneously reducing bias in the measured B_1_^+^ due to T_1_ and magnetization history. The mean coefficient of variation was reduced by 81% for T_1_s of 0.5–3 s compared to conventional satTFL. In vivo, the reproducibility coefficient for flip angles in the range 0–130° was 4.5° for satTFL and 4.7° for our scheme, significantly smaller than for a short TR satTFL sequence, which was 12°. The 3D sequence measured B_1_^+^ maps of the whole thorax in 26 heartbeats.

**Conclusion:**

Our adaptations enable faster B_1_^+^ mapping, with minimal T_1_ sensitivity and lower sensitivity to magnetization history, enabling single breath‐hold whole‐heart absolute B_1_^+^ mapping.

## 1 INTRODUCTION

Ultrahigh field MRI promises superior SNR over conventional clinical MR systems.^1^ However, inhomogeneity in the transmit field (B_1_^+^), due to the wavelength associated with the increased Larmor frequency, produces altered image contrast and significant signal dropout. Various approaches of overcoming this B_1_^+^ inhomogeneity have been proposed in the literature. These include, but are not limited to, the use of adiabatic pulses,^2^ parallel transmit^3^, ^4^ (pTx) methods such as k_T_‐points,^5^ 2D spokes,^6^ and time‐interleaved acquisition of modes.^7^, ^8^ Parallel transmit enables spatial and temporal RF‐shimming overcoming local B_1_^+^ inhomogeneity, but prior knowledge of the channel‐wise B_1_^+^ is required for the optimal RF pulse design. B_1_^+^ mapping at ultrahigh field is more challenging than at lower fields due to the increased dynamic range across an FOV and the limited range of flip angles over which methods are accurate.^9^ Additionally, the acquisition duration increases with the number of transmit channels, necessitating faster mapping schemes. In vivo imaging requires fast and robust B_1_^+^ calibration; however, current methods suffer from limitations of the specific absorption rate (SAR) and inaccuracy due to T_1_ relaxation times.

There is currently no gold standard for in vivo B_1_^+^ mapping, particularly for the heart, where this is compounded by cardiorespiratory motion. Actual flip angle imaging^10^, ^11^ is accurate as well as being insensitive to T_1_ but requires efficient spoiling to eliminate unwanted signal from residual transverse magnetization. Actual flip angle imaging employs a dual‐TR steady‐state gradient‐refocused echo acquisition and consequently requires many dummy TRs before imaging to ensure operation in a steady state. Bloch‐Siegert shift^12^ is phase‐based and has been shown to obtain motion‐robust cardiac B_1_^+^ maps at 3 Tesla^13^ but suffers from high SAR at ultrahigh field and is affected by susceptibility. Dual refocusing echo acquisition mode (DREAM)^14^ offers an improvement in speed and robustness to motion by acquiring 2 images quasi‐simultaneously using a FID and stimulated echo combination.^15^ DREAM is fast, has low SAR, and in practice has a dynamic range of 7 (10–70°). Although it performs well even in the myocardium, DREAM suffers from dropout in the blood pool of the heart due to flow sensitivity.^16^, ^17^, ^18^ Saturation‐prepared 2 rapid gradient echo (SA2RAGE)^19^ has a large dynamic range of 10 but requires long delay times between 2 TurboFLASH readouts following a saturation pulse, making it unsuitable for cardiac imaging.

B_1_^+^ mapping using presaturation TurboFLASH (satTFL)^20^, ^21^ is extremely SAR‐efficient,^22^ flow‐insensitive, and intrinsically robust to motion. However, it requires long TRs to allow for full T_1_ relaxation between a reference and magnetization‐prepared acquisition. Moreover, satTFL suffers from T_1_ sensitivity due to relaxation during the TurboFLASH readout train. Chung et al.^21^ proposed reducing the time delay (TD) of satTFL down to approximately T_1_, at the cost of reduced SNR, by implementing a “saturation recovery module” using adiabatic pulses to produce a consistent magnetization state between acquisitions. However, this requires uniform saturation across the whole subject, which is difficult to achieve in practice and highly SAR intensive at 7 T.

The aim of this work was to design and evaluate a novel adaptation to satTFL to mitigate the T_1_ sensitivity and shorten the acquisition time, while keeping all the beneficial characteristics typical of B_1_^+^ mapping using satTFL. To achieve this, we “sandwich” the reference and prepared images together, thus removing the 5 T_1_ time delay completely and acquiring these images back‐to‐back.^23^ We evaluate this adaptation in a segmented approach to k‐space acquisition, multi‐transmit flip angle mapping, and as a 3D sequence. This method, we term Sandwich, is compared to a satTFL and short TR satTFL method in simulation, phantoms, and in vivo at 7 T for the brain and heart.

## 2 THEORY

**FIGURE 1:**
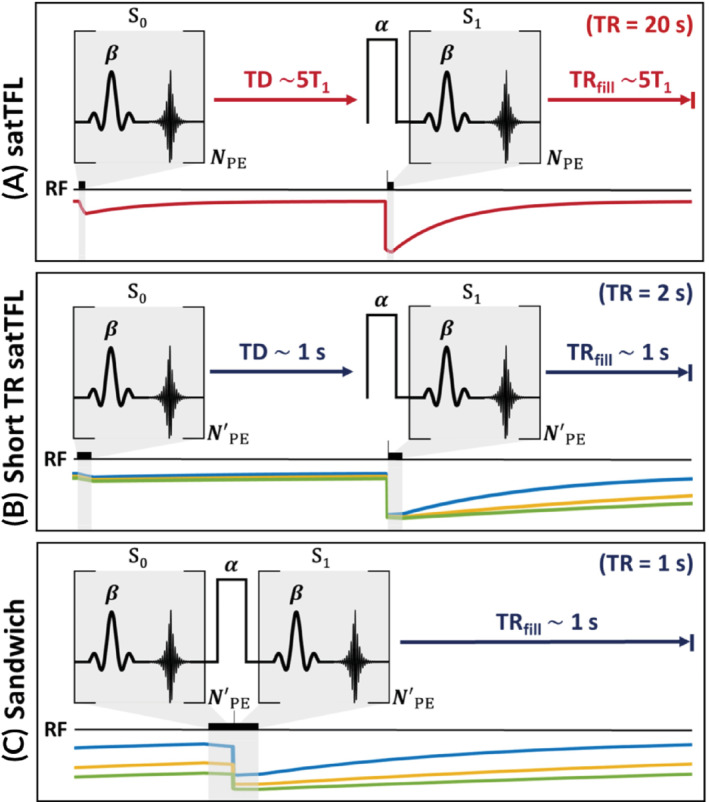
Simplified pulse sequence diagrams of satTFL and the adapted schemes using a preparation pulse flip angle, α, and IT flip angles, β, for a single TR, along with 3 example T_1_ recovery curves (short T_1_ blue, mid‐T_1_ yellow, long T_1_ green) not to scale. (A) satTFL with a long relaxation time between a reference and prepared IT (with *N* phase encodes) allowing for full T_1_ relaxation. (B) Short TR: IT acquired segmented in 4 (with N′ phase encodes; reference images ($S_{0}$) first followed by prepared images ($S_{1}$)) with TD of approximately 1 s and TR of 2 s (C) Sandwiching: IT segmented into 4 shots and TD minimized, images acquired pairwise. Square brackets indicate a TurboFLASH repetition period (TR_FLASH_) for phase encoding. Each phase‐encoding step and preparation RF pulse is followed by gradient spoiling in addition to RF spoiling. Note (B) and (C) start from incomplete recovery of the longitudinal magnetization due to prior TRs. IT, image train; satTFL, presaturation TurboFLASH; TD, time delay; TR_FLASH_, TurboFLASH repetition period.

The satTFL B_1_^+^ mapping sequence^20^ measures a series of rapid FLASH images, each with a different magnitude preparation pulse. Chung et al.^21^ demonstrated that only 2 FLASH images are needed: 1 reference ($S_{0}$) and 1 magnetization‐prepared ($S_{1}$) with a nominal preparation flip angle of α (typically of order 60–120°), as depicted in Figure 1A. The flip angle of the preparation pulse is determined as,

(1) $$\alpha =\arccos\left(\frac{S_{1}}{S_{0}}\right).$$

Chung et al.^21^ recommended having a TD between $S_{0}$ and $S_{1}$ to enable full relaxation or using a “reset pulse” to image faster because reducing this delay introduces a T_1_ dependence, as shown in Figure 1B. In this work, we evaluate an alternative approach to enable a much faster satTFL acquisition. We set TD = ∼0 s such that the total duration of $S_{0}$ and $S_{1}$ is much shorter than T_1_. This results in the T_1_ contrast in $S_{0}$ being matched to $S_{1}$ as depicted in Figure 1C. The matched T_1_ contrast removes the sensitivity to T_1_ and enables the use of Equation 1 to calculate the flip angle. We refer to this timing as the sandwich scheme. In our experiments we found that the image excitation (β) should be subjected to the same B_1_^+^ field as the preparation pulse α, and in our sandwich scheme experiments below they are linearly proportional.

**FIGURE 2:**
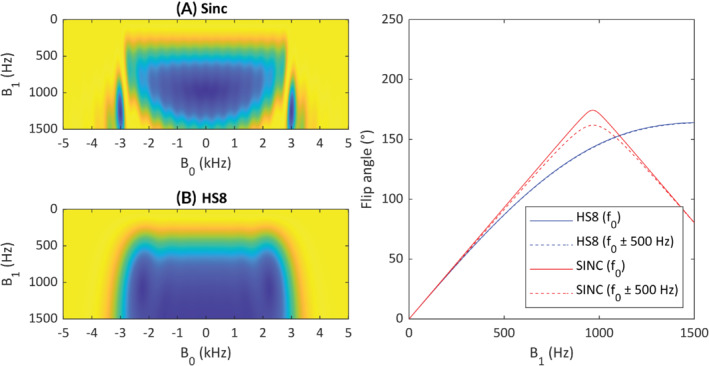
Bloch simulation of (A) sinc pulse (5 ms) and (B) HS8 (5 ms) pulses used for saturation of the magnetization‐prepared image. HS8 pulse is highly insensitive to changes in B_0_ as well as 20% of the peak B_1_^+^ compared to the equivalent sinc pulse. Correction for the nonlinearity of the HS8 pulse response to B_1_^+^ must be performed. HS8, hyperbolic secant.

Due to the large B_0_ inhomogeneities in a 3D volume at 7 T, we opted to use a 5 ms nonselective broad‐band full‐passage hyperbolic secant (HS8) pulse (time‐bandwidth product of 15), operating below the adiabatic limit, as our preparation pulse^24^ in the 3D schemes (Figure 2). The HS8 pulse is insensitive to B_0_ over a broad frequency range determined by the time‐bandwidth product. Additionally, the HS8 pulse offers a low peak RF voltage, making it suited to regions of low B_1_^+^ and high B_0_ inhomogeneity.

## 3 METHODS

### 3.1 Numerical simulations

**FIGURE 3:**
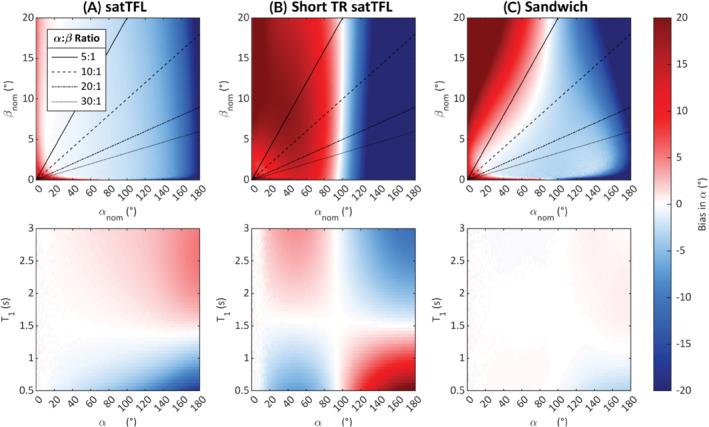
The effects of a nominally applied preparation pulse, $\alpha_{\mathrm{nom}}$, and image train flip angle, $\beta_{\mathrm{nom}}$, on the mean bias in the flip angle map (top row) and the effect of T_1_ on the measured flip angle (bottom row) for the 3 different 2D B_1_^+^ mapping sequences (A) satTFL (B) short TR satTFL (C) sandwich. Simulations performed using a T_1_ of 2 s (top row) and fixed α:β = 10:1 (bottom row) with a fixed noise level added across all schemes using a Monte Carlo method with 1000 repeats, SD in Supporting Figure S1. Dark red signifies large positive overestimation of *α* and dark‐blue large underestimations. Four lines of constant preparation pulse to image train flip angle (α:β) ratio are plotted and shown in the common legend in (A). (bias >20° is color saturated to give a greater dynamic color range to bias in range of interest, i.e., in range −20° to 20°). satTFL, presaturation TurboFLASH.

**FIGURE 4:**
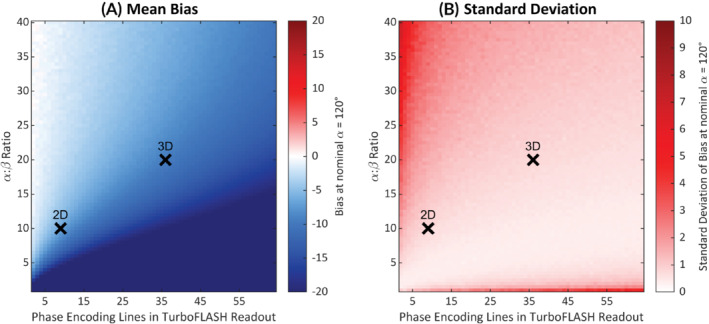
Bias (A) and SD (B) at different α:β ratios and number of phase‐encoding lines within a TurboFLASH readout (*N*′) shown for a nominal α of 120°. The parameters, which correspond to the 2D (α:β = 10:1, *N*′ = 9) and 3D (α:β = 20:1, *N*′ = 36) sequences used in this paper, are marked by crosses.

All sequences were simulated using the extended phase graph formalism^25^ in MatLab (R2021a, MathWorks, Natick, MA) to explore the effects of α, β, TR between segments, T_1_, and the length of the TurboFLASH imaging train ($N^{\prime }$) on the measured B_1_^+^ for each method. The effect of noise was investigated using Monte Carlo simulation. Nominal flip angles $\alpha_{\mathrm{nom}}$ and $\beta_{\mathrm{nom}}$ were varied from 1–180° and 1–20°, respectively. Similarly, the effect of T_1_ relaxation on the calculated value of α was investigated by simulating a range of T_1_ times (0.5–3 s). Simulation details can be found in the Supporting Information, and figure‐generating code for Figures 2, 3, 4 and S1 can be found at https://github.com/jameslewiskent/sat2TFL.

### 3.2 Parallel transmit MRI system

A 7 T MRI (Magnetom VB17, Siemens Healthineers, Erlangen, Germany) equipped with parallel transmit was used for phantom and in vivo imaging. Phantom and brain images were acquired using the same 8‐channel transmit, 32‐channel receive head coil (Nova Medical, Wilmington MA) in circularly polarized (CP) mode at magnet isocentre. To image the heart, an 8‐channel transmit/receive dipole array (MR Coils BV, Zaltbommel, Netherlands) was used with cardiac triggering using a pulse oximeter.

### 3.3 Sequence implementation

On the scanner, 2D acquisitions used an α:β ratio of 10:1, based on our simulations, with nominal α/β flip angles 90/9°. 2D B_1_^+^ maps were measured at 4 different reference voltages of 30, 60, 90, and 120 V, achieving α flip angles of approximately 40°, 80°, 120°, and 160° in the center of the brain to assess the linearity and the dynamic range. K‐space was segmented into 4 shots (readout trains) for all but the conventional satTFL method, each with $N^{\prime }$ = 9 phase‐encoding lines. The TD between consecutive images and the TR to acquire the pair of $S_{0}$ and $S_{1}$ images per segment (TD/TR) were 9.85/20 s for satTFL and 0.96/2 s for short TR satTFL, with TR_fill_ = TD. The sandwich method had a TD/TR of 0/1 s, with a TR_fill_ of 0.92 s. TurboFLASH TE 1.78 ms and TurboFLASH TR (TR_FLASH_) 4.2 ms, FOV = 250 × 250 mm^2^, slice thickness = 8 mm, scan matrix = 50 × 36 (including asymmetric echo, 75% partial Fourier, and 75% phase resolution), bandwidth = 490 Hz/px, centric phase‐encoding, and RF spoiling. B_1_^+^ information is encoded using a 0.5 ms nonselective rectangular pulse, α, and imaging with a sinc pulse, β. The short TR satTFL sequence acquires the 4 segments of $S_{0}$, followed by the 4 segments of $S_{1}$, unlike the sandwich scheme where these segmented images are acquired pairwise. Additionally, we implement 2 dummy TR periods for our sandwich scheme prior to data acquisition, ensuring consistent magnetization. A reference B_1_^+^ map was acquired using a segmented (4 segments with a readout duration of 40 ms) satTFL map with TD/TR = 9.96/20 s. The shorter imaging train used is expected to minimize the T_1_ sensitivity of the satTFL method. The reference map was acquired at 2 voltage levels (60 and 90 V) to obtain a best estimate of the ground truth, and a mean reference map was calculated for comparison to the other methods. The acquisition time was 80 s for the reference scheme, 20 s for satTFL, 8 s for short TR satTFL, and 6 s (including 2 dummy TR periods) for the sandwich scheme.

3D B_1_^+^ maps were also acquired with k‐space segmented into 18 (brain) or 24 (heart) shots of $N^{\prime }$ = 36 phase‐encoding lines per shot, which is 1 partition per TR. The scan matrix was 50 × 36 × 18/24 (brain/heart). Nonselective excitation with a TurboFLASH TE/TR_FLASH_ = 0.87/2.7 ms was used, and a HS8 pulse as our preparation pulse. Based on simulations, we determined that for the longer TurboFLASH image train of the 3D sequences, a higher α:β ratio of 20:1, using nominal α/β flip angles 130/6.5°, was preferable and improves the linear dynamic range.

### 3.4 Image reconstruction

Image reconstruction was performed offline in MatLab (R2021a, MathWorks). The k‐space data were zero‐padded to 64 × 64 (2D) or 64 × 64 × 18/24 (3D brain/heart), and a Hanning filter was applied to reduce Gibbs ringing. Separate receive channel images were then formed from a fast Fourier transform and combined using coil sensitivity profiles calculated from ESPIRiT^26^ to produce complex images $S_{0}$ and $S_{1}$. The resulting maps were then calculated using Equation (1). The B_1_^+^ maps in phantom and in vivo were masked using a simple value‐thresholding approach informed from the reference image and slightly eroded, except in the 3D cases where masks were manually drawn.

### 3.5 Phantom experiments

Two spherical phantoms containing silicon oil/water were used. In addition, 2D individual pTx and 3D B_1_^+^ maps were obtained for each method.

### 3.6 In vivo experiments

Six healthy volunteers were scanned in accordance with our institution's ethical practices. 2D B_1_^+^ maps were measured in the head using each of the methods for 4 different reference voltages. Voxel‐wise correlation and Bland–Altman plots compare each method to a reference map.

A benefit of the sandwich method is that the paired acquisition of a reference and prepared image provides insensitivity to magnetization history because each $S_{1}$ image has a matching $S_{0}$ image. This facilitates rapid multi‐transmit mapping because you can switch to a different B_1_^+^ shim without waiting for full relaxation of the longitudinal magnetization. In 1 volunteer, the sandwich scheme was evaluated for rapid 2D single‐shot (nonsegmented, readout duration of 151 ms) B_1_^+^ mapping of individual transmit channels in the brain and compared to satTFL and short TR satTFL. Due to the longer readout train, a higher α:β ratio was used of 20:1, using nominal α/β flip angles 130/6.5°. The satTFL schemes acquired 1 $S_{0}$ image and 8 $S_{1}$ images transmitting with β in CP+ mode and α 1 channel at a time. The acquisition time was 90 s for satTFL and 9 s for short TR satTFL scheme. For our sandwich scheme, to ensure a constant α:β ratio (within a TR period), the same B_1_^+^ shim was used for both α and β. RF pulses were transmitted 1 channel at a time for an acquisition time of 8 s; no dummy pulses were performed because the B_1_^+^ shim changes between TR periods. In addition to these absolute maps, an interleaved coil‐cycled gradient echo sequence was performed to obtain relative maps and used to rephase and weight the total B_1_^+^. To evaluate the single channel maps, we combined them using the phase of relative transmit maps to form a synthetic CP mode map and compared the result to a map measured in CP mode. The acquisition parameters for the interleaved coil‐cycled gradient echo sequence to obtain these relative maps were: matched FOV and 64 × 64 scan matrix, flip angle 7°, TE/TR_GRE_ = 1.9/4.3 ms for a total acquisition time of 2 s.

In another healthy volunteer, the sandwich scheme was evaluated as a 3D B_1_^+^ mapping acquisition in the brain and compared to satTFL and short TR satTFL. A scan matrix of 50 × 36 × 18 was used with a FOV of 250 × 250 × 180 mm^3^, 10 mm partition thickness, with a reference voltage of 60 V. For the satTFL methods, the TD/TR was 9.9/20 s for satTFL and 0.9/2 s for short TR satTFL, with TR_fill_ = TD. The sandwich method had a TD/TR of 0/1 s, with a TR_fill_ of 0.8 s. The total acquisition time for satTFL was 360 s, the short TR satTFL scheme took 36 s, and the sandwich scheme took 20 s (including 2 dummy TRs).

Lastly, to evaluate the sandwich scheme for whole‐heart, single breath‐hold, B_1_^+^ mapping a 3D sandwich B_1_^+^ map was measured in the heart of a healthy volunteer. For reference, a 2D satTFL B_1_^+^ map was acquired along the horizontal long axis of the heart. The acquisition parameters for the 3D sandwich were a scan matrix of 50 × 36 × 24 with a FOV of 384 × 384 × 240 mm^3^, 10 mm partition thickness, and a 200 V reference voltage. The total acquisition time was 20 heartbeats for a single 2D satTFL B_1_^+^ map (minimum TD = 10 s) and 26 heartbeats for the 3D sandwich scheme, each acquired within a breath‐hold. A slice through the 3D sandwich data was oriented and resampled, using a linear interpolation in the FMRIB Software Library (FSL),^27^ to match the 2D satTFL for comparison.

## 4 RESULTS

The FWHM of the point spread function indicates intervoxel blurring is low for all schemes across various T_1_ times. Figure 3 shows the mean bias for different simulated nominal α and β and the effect of T_1_ on the measured value of α. As expected, satTFL exhibits relatively low bias in α and the least sensitivity to noise (Supporting Figure S1) due to the long TD of 5 T_1_ between the reference and prepared images. The satTFL scheme also shows little variation in bias for different ratios of α:β, which is not the case for the other schemes. Figure 3B reports large under‐ and overestimations for short TR satTFL across a range of preparation pulse flip angles for all nominal α:β ratios, which is also T_1_‐dependent. This error in α is due to there being insufficient time for the longitudinal magnetization to fully relax to equilibrium. As a result, the longitudinal magnetization is not consistent prior to imaging. Figure 3C shows the sandwich scheme performs remarkably well using a TR of 1 s due to the segmentation and “sandwiching” of the 2 image trains. The bottom row of Figure 3 shows that at a fixed α:β ratio of 10:1 there is some T_1_‐dependent bias introduced, which increases with the preparation flip angle for both satTFL schemes. However, this bias due to T_1_ is greatly reduced for the sandwich scheme. Contrastingly, noise is more significant in the sandwich scheme (Supporting Figure S1) due to the reduced longitudinal magnetization in the steady state and depleted magnetization following $S_{0}$ (resulting in a lower SNR $S_{1}$). We identify that using an α:β ratio of 10:1 may yield the best compromise between a large range of low‐bias *α* and low sensitivity to noise for 2D measurements; thus, all 2D experiments (except for 2D multi‐transmit mapping) use this ratio.

The effect of the readout train length and α:β ratio on the mean bias and SD is shown in Figure 4. Higher α:β ratios are likely to be beneficial for 3D sequences where the length of the TurboFLASH imaging trains is longer.

**FIGURE 5:**
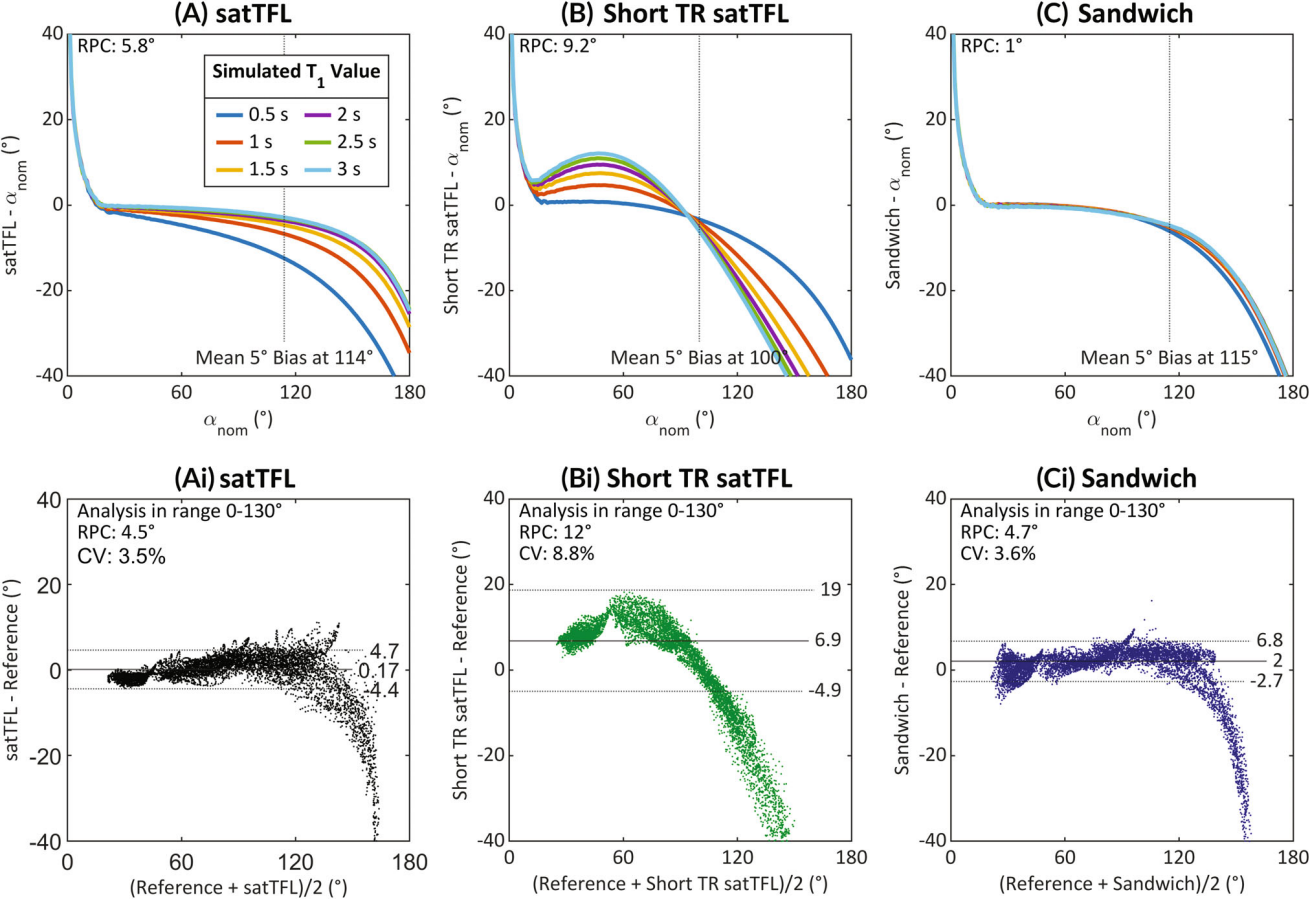
Simulation results for various T_1_ values (0.5–3 s) (top row) for each scheme (A) satTFL (B) short TR satTFL and (C) sandwich and Bland–Altman plots for in vivo 2D brain data (bottom row). Simulation shows the insensitivity of our sandwich scheme to variation in T_1_. The Bland–Altman plots show data aggregated from 5 volunteers for 4 B_1_^+^ maps at reference voltages of 30, 60, 90, and 120 V for greater dynamic range. The 120 V datasets are removed from the Bland–Altman analysis due to oversaturation beyond the linear region but remain in the figure to show the response. Dotted lines in Bland–Altman plots represent ±1.96 SDs from the mean. satTFL, presaturation TurboFLASH.

The top row in Figure 5 shows simulation results for various T_1_ values, and the bottom row shows Bland–Altman plots of measurements for each scheme from 2D B_1_^+^ maps acquired at 4 reference voltages to cover a larger dynamic range. For both satTFL and short TR satTFL, short T_1_'s result in a spread of measured flip angles deviating by up to 12° compared to a long T_1_ (evaluated at 120°), whereas this spread is reduced to <2° with the sandwich scheme. The short TR satTFL scheme shows especially large T_1_‐dependent bias. There is good agreement between the sandwich and reference methods. However, the limits of agreement vary with α and the sandwich scheme average measured α deviates >5° above a nominal α of 115°. A correlation plot for 1 subject can be seen in Supporting Figure S2 combined with simulation.

**FIGURE 6:**
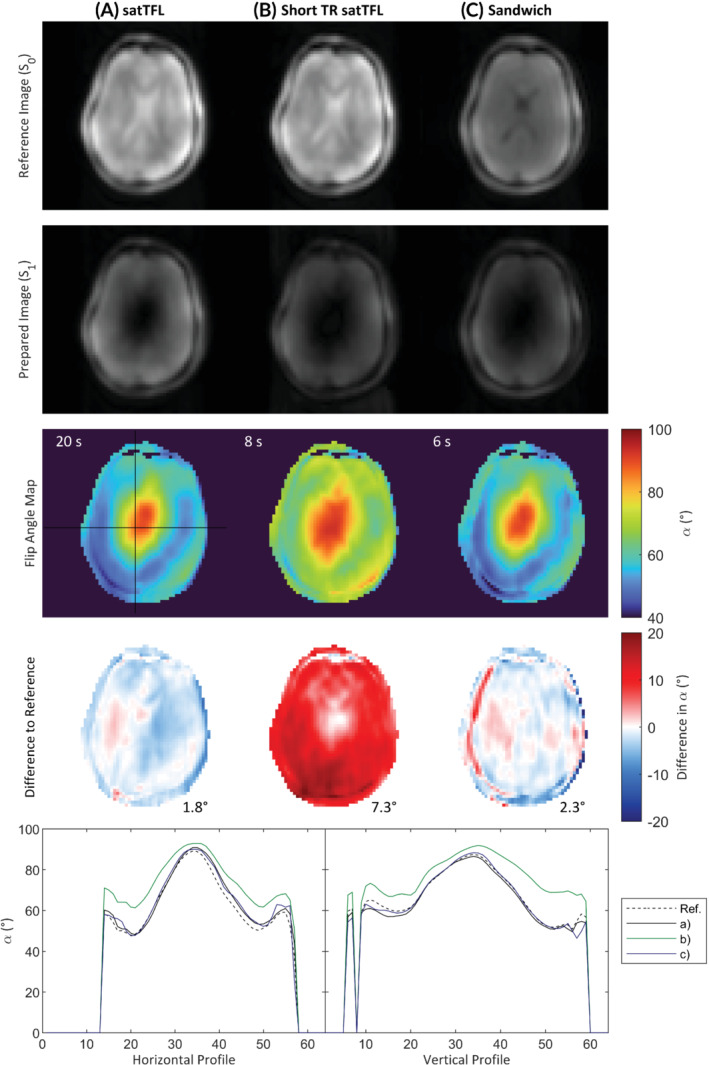
In vivo brain B_1_^+^ maps of 1 volunteer at reference voltage 60 V for various 2D sequences. (A) satTFL (B) segmented short TR (2 s) satTFL (C) segmented sandwiched scheme with TR = 1 s, α:β = 10:1. The first 2 rows show the magnitude of the images ($S_{0}$ and $S_{1}$) used to calculate the corresponding flip angle maps in the third row. The difference between each of the maps at 60 V and a mean reference map (calculated from 2 reference voltages for the reference scheme and scaled by the voltage) are shown in the fourth row, along with the RMSE. Flip angle profiles for horizontal and vertical profiles through the center of the brain are also shown. Note the reduced time to acquire (C) was 6 s compared to 20 s for (A) and 8 s for (B). satTFL, presaturation TurboFLASH.

Figure 6 shows 2D flip angle maps of an axial slice of the brain from a single volunteer for each sequence at a reference voltage of 60 V. The first and second rows show the magnitude of the $S_{0}$ and $S_{1}$ images. As expected, the SNR associated with the sandwich method (Figure 6C) has approximately halved in both $S_{0}$ and $S_{1}$ compared to satTFL. All maps show qualitatively similar results and large B_1_^+^ inhomogeneity, with the expected brightening around the region of the lateral ventricles due to imaging in CP mode. Structural contrast is only visible in the B_1_^+^ maps of short TR satTFL. The difference between the reference map and the maps acquired with the other schemes is shown in the fourth row along with the RMSE. Comparable results in phantoms are shown in the Supporting Figures S3 and S4.

**FIGURE 7:**
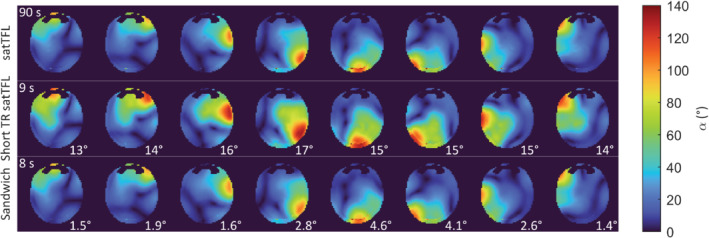
Single‐channel 2D in vivo B_1_^+^ maps for an axial slice through the brain of a single volunteer for an 8Tx‐32Rx head coil. Top row shows individual maps for the satTFL scheme with a TD = 10 s for a total acquisition time of 90 s (1 reference [β in CP+ mode] and 8 prepared images [β in CP+ mode and α on 1 channel at a time]). The second row shows individual channel maps for short TR satTFL with a TD of 1 s, for a total acquisition time of 9 s. The third row shows the sandwich scheme (nonsegmented, *N*′ = 36) with a TR = 1 s for a total acquisition time of 8 s [α and β 1 channel at a time]. The RMSE between each channel map and the satTFL scheme are shown in the bottom right‐hand corner. Acquisition time does not include an additional 2 s to acquire relative maps. Rx, receiver;Tx, transmitter; satTFL, presaturation TurboFLASH.

2D pTx maps are shown in Figure 7 for each method. As expected, short TR satTFL displays severe overestimation with an average RMSE to conventional satTFL of 15°. The sandwich displays similar B_1_^+^ transmit maps to satTFL with an average RMSE of less than 3° and acquired in 8 s.

**FIGURE 8:**
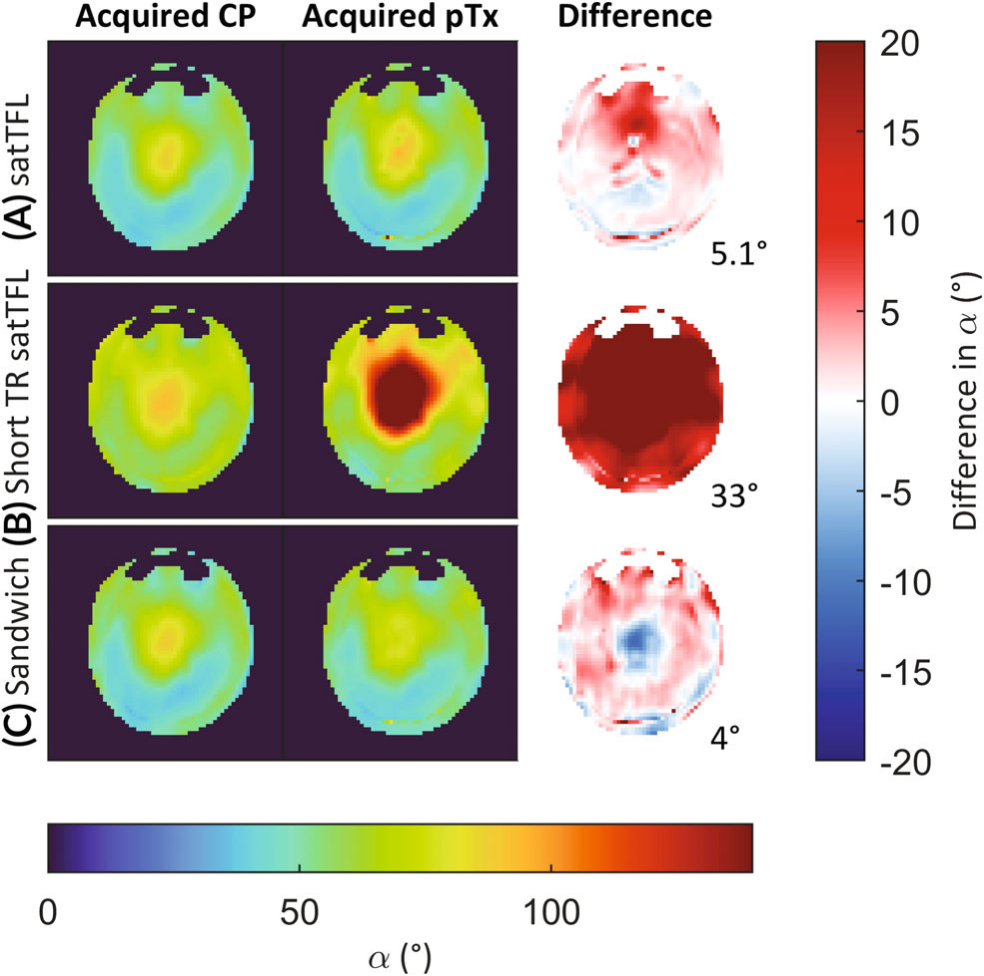
Comparison between acquired CP mode flip angle map and an (8‐channel) pTx map in the brain for (A) satTFL (B) short TR satTFL and (C) sandwich schemes. CP, circularly polarized; pTx, parallel transmit; satTFL, presaturation TurboFLASH.

The difference between an acquired CP mode map to a pTx combined map is shown in Figure 8. The sandwich scheme appears to underestimate the flip angle toward the center of the brain when compared to the acquired CP map, whereas satTFL overestimates. Supporting Figures S5 and S6 show similar results in phantoms. There is good agreement between the acquired and combined CP maps; in the water phantom the RMSE is below 3° for satTFL and 4° for the sandwich scheme.

**FIGURE 9:**
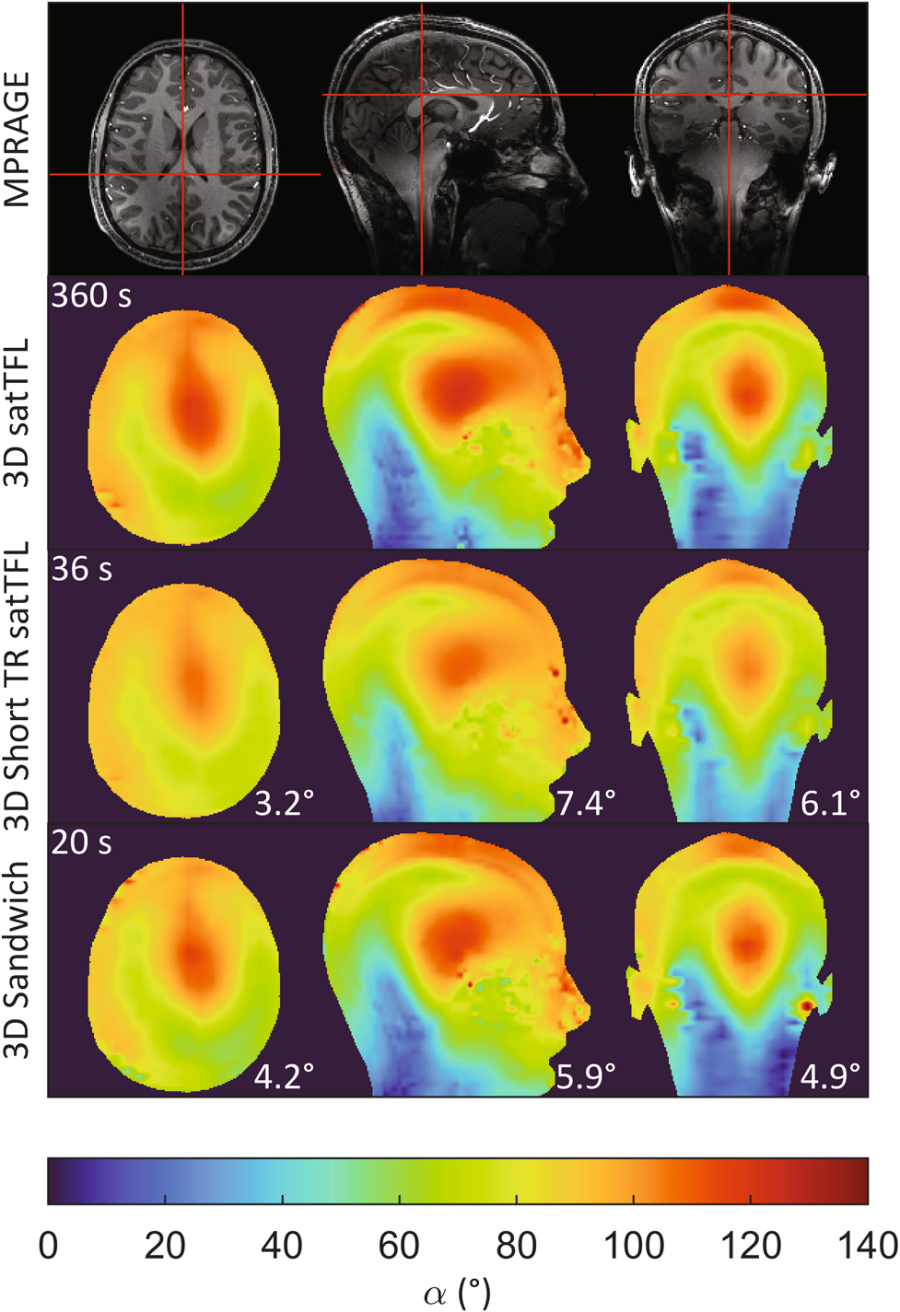
Axial, sagittal, and coronal slices through 3D B_1_^+^ maps for the brain of a volunteer acquired using each method along with a T_1_‐weighted MPRAGE. 3D data acquired in 20 s (including 2 dummy TRs) for our sandwich scheme, 36 s for short TR satTFL, and 360 s for satTFL. B_1_^+^ maps resampled with a linear interpolation using FSL to match the MPRAGE resolution. The RMSE between map and the satTFL scheme are shown in the bottom right‐hand corner. satTFL, presaturation TurboFLASH; FSL, FMRIB software library.

The 3D flip angle maps acquired for 18 partitions in the brain of a volunteer are shown in Figure 9. 3D results in a water phantom are shown in Supporting Figure S7, with an average RMSE of 2.3° over all partitions to satTFL, acquired in 20 s compared to 360 s.

**FIGURE 10:**
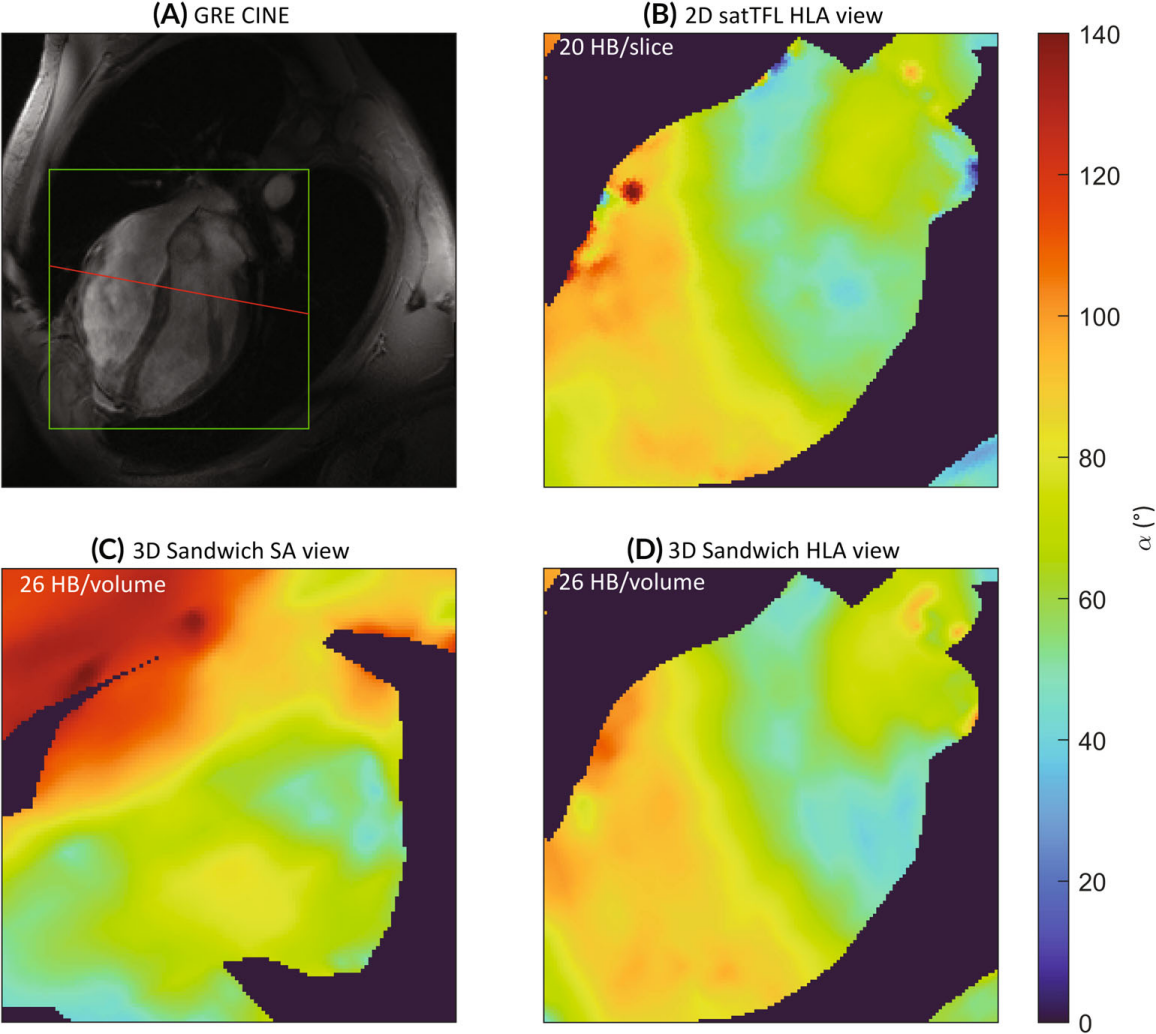
B_1_^+^ maps in the heart of a volunteer acquired using the 3D sandwich sequence in CP mode. A frame from a 2D GRE CINE is shown (A) with the region of interest in green and the red line showing the SA slice through the heart. 3D data acquired in 26 heartbeats (including 2 dummy TRs) for our sandwich scheme is shown along the SA (C) and HLA (D) compared to 20 heartbeats for a single conventional 2D satTFL B_1_^+^ map in (B). The 3D sandwich dataset has been orientated and resampled to match the HLA view of the 2D satTFL in FSL. All images acquired during breath‐hold and gated. GRE, gradient echo; HLA, horizontal long axis; SA, short axis; FSL, FMRIB software library.

Figure 10 shows 3D B_1_^+^ maps acquired in the heart for the sandwich scheme with a 2D satTFL slice along the horizontal long axis of the heart. The high‐resolution CINE demonstrates the inhomogeneous image contrast across the heart at 7 T.

## 5 DISCUSSION

Our simulations (Figure 3) show that removing the delay between $S_{0}$ and $S_{1}$ provides a considerable decrease in acquisition time without substantial loss in the performance for short image trains. Noise is the dominant cause of error at low α, and data acquired in the high‐flip angle region using satTFL shows the most sensitivity to T_1_. This is demonstrated by the increase in spread of measured flip angles. This bias due to T_1_ in the satTFL sequence cannot be corrected for unless the local T_1_ is known. Figure 3B conveys why shortening of the satTFL TR period is unwise because it introduces significant T_1_‐dependent bias. The sandwich scheme, as expected from simulations, shows a reduced T_1_ sensitivity. The sandwich scheme also performs similarly well to satTFL in the range 40–120°. On the other hand, compared to satTFL, the dynamic range is slightly reduced in our scheme, and we must employ 2 dummy TRs to ensure consistent magnetization between partitions. The maximum measurable α shifts from 180° for satTFL to approximately 160° for the sandwich scheme. However, satTFL is rather inaccurate when measuring at this extreme due to T_1_.

The simple arccosine model holds when both β is small and the readout train short; if one is increased the other should be reduced, this is demonstrated in Figure 4. Because the 3D acquisition used a longer readout train, the α:β ratio was increased to 20. This maintained a low T_1_ bias and the dynamic range at the expense of a lower SNR. The SNR loss due to this higher ratio, however, is compensated by the nature of the 3D acquisition. In both 2D and 3D cases, the sandwich scheme departs from the arccosine relationship (Equation 1) at large α; this can be mitigated by replacement with a lookup table.

The discussed novel method has been proven to obtain in vivo maps, which perform similarly to the satTFL method with a significant decrease in acquisition time and less sensitivity to T_1_, as shown in Figure 5. The segmented short TR satTFL method in Figure 6 does not show the same SNR loss as the sandwich scheme because the segmented $S_{0}$ is fully acquired before the segmented $S_{1}$. CSF in the ventricles appears dark in $S_{0}$ of the sandwich scheme due to the long T_1_, unlike satTFL where it appears bright. As seen in Figure 6, for a further reduced scan time than short TR satTFL, the sandwich scheme has only 0.5° higher RMSE than satTFL, largely due to chemical shift effects and remains accurate within the brain.

A benefit of the paired acquisition of a reference and prepared image provides insensitivity to magnetization history, which also enables rapid multi‐transmit mapping. 2D single‐transmit maps were acquired for each scheme, as shown in Figure 7. Our sandwich method provided a substantial reduction in acquisition time while maintaining a small RMSE to satTFL. The pTx combined map in Figure 8 shows that the sandwich scheme tended to underestimate at the center of the brain, whereas satTFL tended to overestimate, which was also seen in Supporting Figure S6 for the water phantom. However, the satTFL pTx acquired map in Figure 8 shows much larger overestimation than seen in phantom and shows structural artifacts likely due to sensitivity to T_1_, not seen for our sandwich scheme. Improvements in the multi‐transmit maps could be achieved using a linear combination of transmit sensitivities, such as Fourier encoding.^28^, ^29^

The 3D sandwich scheme performs similarly well with low RMSE to satTFL (Figure 9), allowing a 3D B_1_^+^ map to be acquired in approximately the same time as a single TR of satTFL (20 s). In Figure 10, both satTFL and the sandwich scheme show similar B_1_^+^ map profiles in the heart. Recent work has applied 3D FLASH readouts to satTFL^30^, ^31^ to enable single‐shot acquisitions of an entire brain volume. Although this works well in the brain, this is not suitable for the heart because the readout is much longer than diastole and T_1_ effects become a concern.

Aside from the 2 additional dummy TR periods, our sandwich method does not necessarily change the energy deposited compared to the conventional satTFL method (Supporting Table S1). Hence, due to the much shorter duration, our method shows a large increase in SAR, which is directly proportional to the reduction in acquisition time. In particular, the use of 2 dummy TR periods and reduction in scan time accounts for approximately a factor of 22‐fold increase in SAR over conventional satTFL. Supporting Table S1 also shows that compared to a matched acquisition DREAM sequence, there is a similar average power deposition for our 3D sandwich scheme. Using a nonselective broad‐band full‐passage HS8 pulse as the preparation pulse does contribute significant additional energy over the standard rectangular pulse, although we are still <10% of power limits. The HS8 has equivalent RF energy but with only 20% of peak B_1_^+^ for an equivalent bandwidth and flip angle sinc pulse, reducing the effect of hardware constraints on deliverable peak B_1_^+^. Additionally, the HS8 is robust to large *B*_0_ inhomogeneities over a bandwidth ±1.5 kHz (Figure 2), while maintaining a monotonic relationship to B_1_^+^. In our experience, the HS8 bandwidth covers the range of B_0_ expected in both the head and the heart. The HS8 pulse, however, is nonlinear for higher flip angles, and a look‐up table correction should be used to account for this. The nonlinearity of the HS8 at high flip angles, with the use of a look‐up table, facilitates an increase in the measurable B_1_^+^ up to 200°.

An adaptation to the commonly used satTFL B_1_^+^ mapping sequence has been introduced, and the performance of the method in simulations and in experiments—both in phantom and in vivo—has been demonstrated. The intention here has been to show the ability to produce absolute volumetric flip angle maps for the purpose of body imaging, with validation in the brain and heart. The adaptations retain the relatively low SAR, are less T_1_‐dependent, require no special saturation pulses, and are more robust to motion, that is, able to obtain $S_{0}$ and $S_{1}$ within the same cardiac cycle.

Sandwiching the images overcomes the issues associated with a short TR satTFL sequence by minimizing T_1_ relaxation between $S_{0}$ and $S_{1}$. Our method achieves similar measurement times as DREAM, but without the flow artifacts, and may be of potential interest for cardiac imaging, as well as having applications elsewhere. Further work could combine these adaptations with methods such as transmit low rank.^32^ Transmit low rank in combination with a B_1_^+^ time interleaved acquisition of modes^8^ style acquisition could allow acceleration of multi‐channel mapping by up to a factor of 8,^33^ and may allow single‐shot volumetric multi‐channel mapping of the pTx array in a single breath‐hold for cardiac gated applications.

## 6 CONCLUSION

The sandwiched presaturation TurboFLASH (Sandwich) sequence enables fast acquisitions with reduced T_1_ bias and facilitates 3D B_1_^+^ measurements of the whole thorax or brain in 26 s or less.

## FUNDING INFORMATION

j.l.k. received support of Engineering and Physical Sciences Research Council (EPSRC) through a Cooperative Awards in Science and Technology (iCASE) award in collaboration with Siemens Healthineers. The Wellcome Center for Integrative Neuroimaging is supported by core funding from the Wellcome Trust, grant (203139/Z/16/Z). l.v. is funded by a Sir Henry Dale Fellowship awarded jointly by the Wellcome Trust and the Royal Society (221805/Z/20/Z) and by the Slovak Grant Agencies VEGA (2/0003/20) and APVV (#19‐0032).

## CONFLICT OF INTEREST

James L. Kent is partially funded by an iCASE stipend award from Siemens Healthineers. Iulius Dragonu is employee of Siemens Healthcare Limited.

## Supporting information


**FIGURE S1:** The effects of a nominally applied preparation pulse, $\alpha_{\mathrm{nom}}$, and image train flip angle, $\beta_{\mathrm{nom}}$, on the standard deviation of the bias in the flip angle map (top row) and the effect of *T*_1_ on the measured flip angle (bottom row) for the three different B_1_^+^ mapping sequences. Simulations performed using a *T*_1_ of 2 s (top row) and fixed α:β=10:1 (bottom row) with a fixed noise level added across all schemes using a Monte Carlo method with 1000 repeats. Four lines of constant preparation pulse to image train flip angle (α:β) ratio are plotted and shown in the common legend in (a).
**FIGURE S2**: Correlation plots for 2D in vivo brain data for (a) satTFL, (b) short TR segmented satTFL and (c) our sandwich scheme plotted against the measured flip angle from the segmented satTFL method acting as reference. The data in each plot are aggregated from four flip angle maps at varying reference voltages (30 , 60, 90 and 120 V) for greater dynamic range, each point representing the flip angle measured in a single voxel from the ROI (shown in top left). The shaded region corresponds to the deviation due to *T*_1_ values in the range 0.5–3 s for a fixed noise level.
**FIGURE S3**: B_1_^+^ maps from a silicon oil phantom at reference voltage 60 V for various sequences. (i) segmented satTFL method with TR = 20 s (ii) satTFL (iii) satTFL acquired segmented and TR reduced to 2 s (iv) segmented sandwiched scheme with TR = 1 s, β=0.1α. The first two rows show the magnitude of the images used to calculate the corresponding flip angle maps in the third row. The difference between each of the maps at 60 V and a mean reference map, calculated from three reference voltages for the reference scheme and scaled by the voltage are shown in the fourth row, along with the RMSE. Flip angle profiles for horizontal and vertical profiles through the center of the phantom are also shown. Note the reduced time to acquire (iv) was 6 s compared to 80 s for (i), 20 s for (ii) and 8 s for (iii).
**FIGURE S4**: Flip angle maps from a water phantom at reference voltage 60 V for various sequences. (i) segmented satTFL method with TR = 20 s (ii) satTFL (iii) satTFL acquired segmented and TR reduced to 2 s (iv) segmented sandwiched scheme with TR = 1 s, β=0.1α. The first two rows show the magnitude of the images used to calculate the corresponding flip angle maps in the third row. The difference between each of the maps at 60 V and a mean reference map, calculated from three reference voltages for the reference scheme and scaled by the voltage are shown in the fourth row, along with the RMSE. Flip angle profiles for horizontal and vertical profiles through the center of the phantom are also shown. Note the reduced time to acquire (iv) was 6 s compared to 80 s for (i), 20 s for (ii) and 8 s for (iii). Note also that the dielectric properties of the water phantom give rise to much more inhomogeneous flip angle profiles than for the (non‐polar) silicon oil phantom.
**FIGURE S5**: Comparison between acquired CP mode flip angle map and an (eight channel) pTx map for a silicon oil phantom.
**FIGURE S6**: Comparison between acquired CP mode flip angle map and an (eight channel) pTx map for a water phantom.
**FIGURE S7**: 3D B_1_^+^ maps for 18 partitions (10 mm) of the water phantom acquired using the 3D sequence in CP mode. On the left are maps acquired using satTFL, short TR satTFL and sandwich schemes for comparison. The difference and corresponding RMSE between satTFL and the other sequences is shown on the right. 3D data acquired in 20 s for our sandwich scheme and 36 s for short TR satTFL whereas this took 360 s with satTFL using a TR of 20 s.
**TABLE S1**: Comparison of 10 s energy and total energy for satTFL, the sandwich scheme and DREAM using a reference voltage of 60 V. Power limits for the Nova head coil (8Tx/32Rx) were 12 W short‐term (10 s average) and 24 W long‐term (6 min average). Sinc and HS8 preparation pulses have matched bandwidths of ±3 kHz. Sinc preparation pulses are likely hardware restricted by peak forward voltage.

## Data Availability

Code for generating simulation results (Figures 2, 3, 4 and Supporting Information S1) is available at https://github.com/jameslewiskent/sat2TFL.
