# Supplementary material for: Rapid 3D absolute B1 + mapping using a sandwiched train presaturated TurboFLASH sequence at 7 T for the brain and heart
Source: Magn Reson Med. 2022 Nov 6;89(3):964–76. doi: 10.1002/mrm.29497 (PMC10099228; doi:10.1002/mrm.29497)
Supplement: Supplementary file 1 — FIGURE S1: The effects of a nominally applied preparation pulse, αnom, and image train flip angle, βnom, on the standard deviation of the bias in the flip angle map (top row) and the effect of T 1 on the measured flip angle (bottom row) for the three different B1 + mapping sequences. Simulations performed using a T 1 of 2 s (top row) and fixed α:β=10:1 (bottom row) with a fixed noise level added across all schemes using a Monte Carlo method with 1000 repeats. Four lines of constant preparation pulse to image train flip angle (α:β) ratio are plotted and shown in the common legend in (a). FIGURE S2: Correlation plots for 2D in vivo brain data for (a) satTFL, (b) short TR segmented satTFL and (c) our sandwich scheme plotted against the measured flip angle from the segmented satTFL method acting as reference. The data in each plot are aggregated from four flip angle maps at varying reference voltages (30 , 60, 90 and 120 V) for greater dynamic range, each point representing the flip angle measured in a single voxel from the ROI (shown in top left). The shaded region corresponds to the deviation due to T 1 values in the range 0.5–3 s for a fixed noise level. FIGURE S3: B1 + maps from a silicon oil phantom at reference voltage 60 V for various sequences. (i) segmented satTFL method with TR = 20 s (ii) satTFL (iii) satTFL acquired segmented and TR reduced to 2 s (iv) segmented sandwiched scheme with TR = 1 s, β=0.1α. The first two rows show the magnitude of the images used to calculate the corresponding flip angle maps in the third row. The difference between each of the maps at 60 V and a mean reference map, calculated from three reference voltages for the reference scheme and scaled by the voltage are shown in the fourth row, along with the RMSE. Flip angle profiles for horizontal and vertical profiles through the center of the phantom are also shown. Note the reduced time to acquire (iv) was 6 s compared to 80 s for (i), 20 s for (ii) and 8 s for (iii). FIGURE S4: Flip [file MRM-89-964-s001.docx]

**Supporting Information for**

‘Rapid 3D Absolute B_1_^+^ Mapping using a Sandwiched Train Pre-Saturated TurboFLASH Sequence at 7T for the Brain and Heart’

James L. Kent^a^, Iulius Dragonu^b^, Ladislav Valkovič^c,d^, and Aaron T. Hess^a^

^a^ Wellcome Centre for Integrative Neuroimaging, FMRIB, Nuffield Department of Clinical Neurosciences, University of Oxford, Oxford, United Kingdom

^b^ Siemens Healthcare Limited, Frimley, United Kingdom

^c^ Oxford Centre for Clinical Magnetic Resonance Research (OCMR), University of Oxford, Oxford, United Kingdom

^d^ Department of Imaging Methods, Institute of Measurement Science, Slovak Academy of Sciences, Bratislava, Slovakia

Details of extended phase graph (EPG) simulation

Simulations were based on Brian Hargreaves EPG code (<http://web.stanford.edu/~bah/software/epg>). Each complete simulated image train consists of 36 phase-encoding steps, image train TE/TR_FLASH_ = 1.78/4.2 ms, low-angle,$\beta_{nom}$, simulating k-space readouts with spoilers throughout the image train to eliminate transverse magnetisation before each imaging RF pulse. The transverse magnetisation immediately following the preparation pulse was effectively spoiled using a quadratically increasing number of ‘unit’ gradients. RF spoiling was also implemented using a linearly increasing phase increment of $\Delta\phi=50^{\circ}$. The pulse schemes were simulated as described in the theory section with two additional dummy TRs to help reach a steady-state magnetisation for the segmented schemes.

Nominal flip angles $\alpha_{\mathrm{nom}}$ and $\beta_{\mathrm{nom}}$ were simulated from 1-180° in 1° increments and 1-20° in 0.1° increments, respectively. The effect of T_1_ relaxation on $\alpha$ was investigated by simulating a range of T_1_ times 0.5-3 s in increments of 50 ms. The T_2_ for all simulations was kept fixed at 25 ms.

The image trains are reordered to mimic a centrically acquired k-space, zero-filled to produce an array of length 64 and then fast Fourier transformed to obtain the point-spread function (PSF). The relative effects of noise across the different schemes were evaluated using a Monte Carlo simulation with 1000 repetitions, with a fixed level of zero-mean complex Gaussian noise added independently to each image train. Following this, $\alpha$ is calculated using the centre of the PSF and Eq. 1 as given in the main note.

Phantom Details

The water phantom was a 16.5 cm spherical MRS ‘Braino’ phantom (General Electric Medical Systems) containing 10 mmol creatine, 3 mmol choline, 5 mmol Lac, 1 mL/L Gd‐DPTA (Magnevist), 12.5 mmol Glu, 7.5 mmol myo‐Ins, 12.5 mmol NAA, 0.1% sodium azide, 56 mmol sodium hydroxide and 50 mmol potassium phosphate monobasic.

The silicon oil phantom was a 16.5 cm sphere containing silicon-based organic polymer (Polydimethylsiloxane, T_1_/T_2_ = 1420/750 ms) which is non-polar and hence has a more uniform excitation profile.

Supporting Results

***
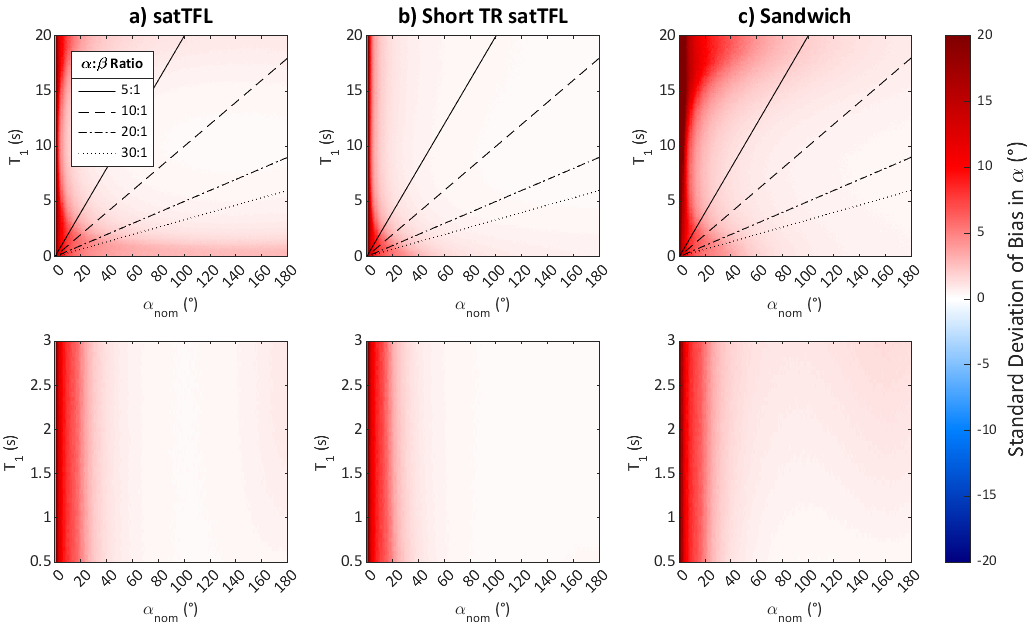
****Figure S1:* The effects of a nominally applied preparation pulse, $\alpha_{\mathrm{nom}}$, and image train flip angle, $\beta_{\mathrm{nom}}$, on the standard deviation of the bias in the flip angle map (top row) and the effect of T_1_ on the measured flip angle (bottom row) for the three different B_1_^+^ mapping sequences. Simulations performed using a T_1_ of 2 s (top row) and fixed $\alpha:\beta=10:1$ (bottom row) with a fixed noise level added across all schemes using a Monte Carlo method with 1000 repeats. Four lines of constant preparation pulse to image train flip angle ($\alpha:\beta$) ratio are plotted and shown in the common legend in a).


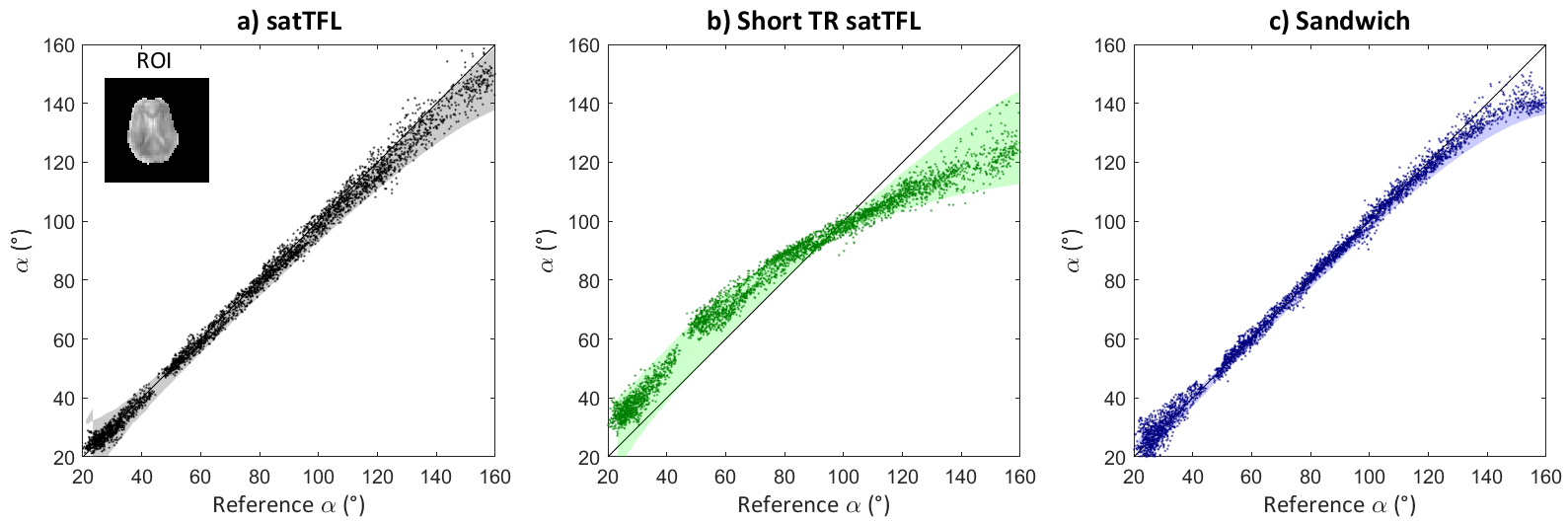
*Figure S2: Correlation plots for 2D in vivo brain data for a) satTFL, b) short TR segmented satTFL and c) our sandwich scheme plotted against the measured flip angle from the segmented satTFL method acting as reference. The data in each plot are aggregated from 4 flip angle maps at varying reference voltages (30 V, 60 V, 90 V and 120 V) for greater dynamic range, each point representing the flip angle measured in a single voxel from the ROI (shown in top left). The shaded region corresponds to the deviation due to T_1_ values in the range 0.5-3 s for a fixed noise level.*


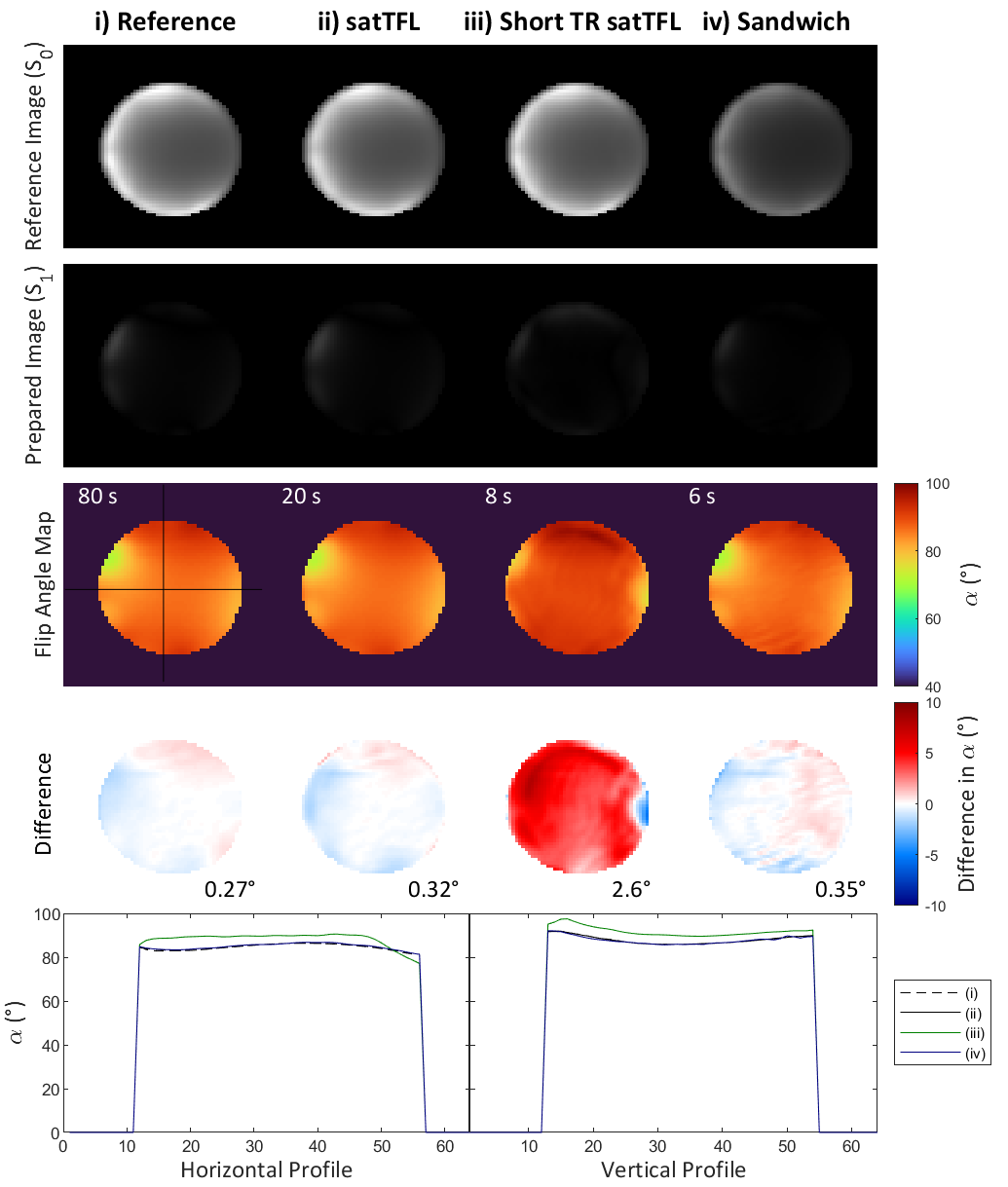
 *Figure S3: B_1_^+^ maps from a silicon oil phantom at reference voltage 60 V for various sequences. i) segmented satTFL method with TR = 20 s ii) satTFL iii) satTFL acquired segmented and TR reduced to 2 s iv) segmented sandwiched scheme with TR = 1 s,* $\beta=0.1\alpha$*. The first two rows show the magnitude of the images used to calculate the corresponding flip angle maps in the third row. The difference between each of the maps at 60 V and a mean reference map, calculated from three reference voltages for the reference scheme and scaled by the voltage are shown in the fourth row, along with the RMSE. Flip angle profiles for horizontal and vertical profiles through the centre of the phantom are also shown. Note the reduced time to acquire iv) was 6 s compared to 80 s for i), 20 s for ii) and 8 s for iii).*


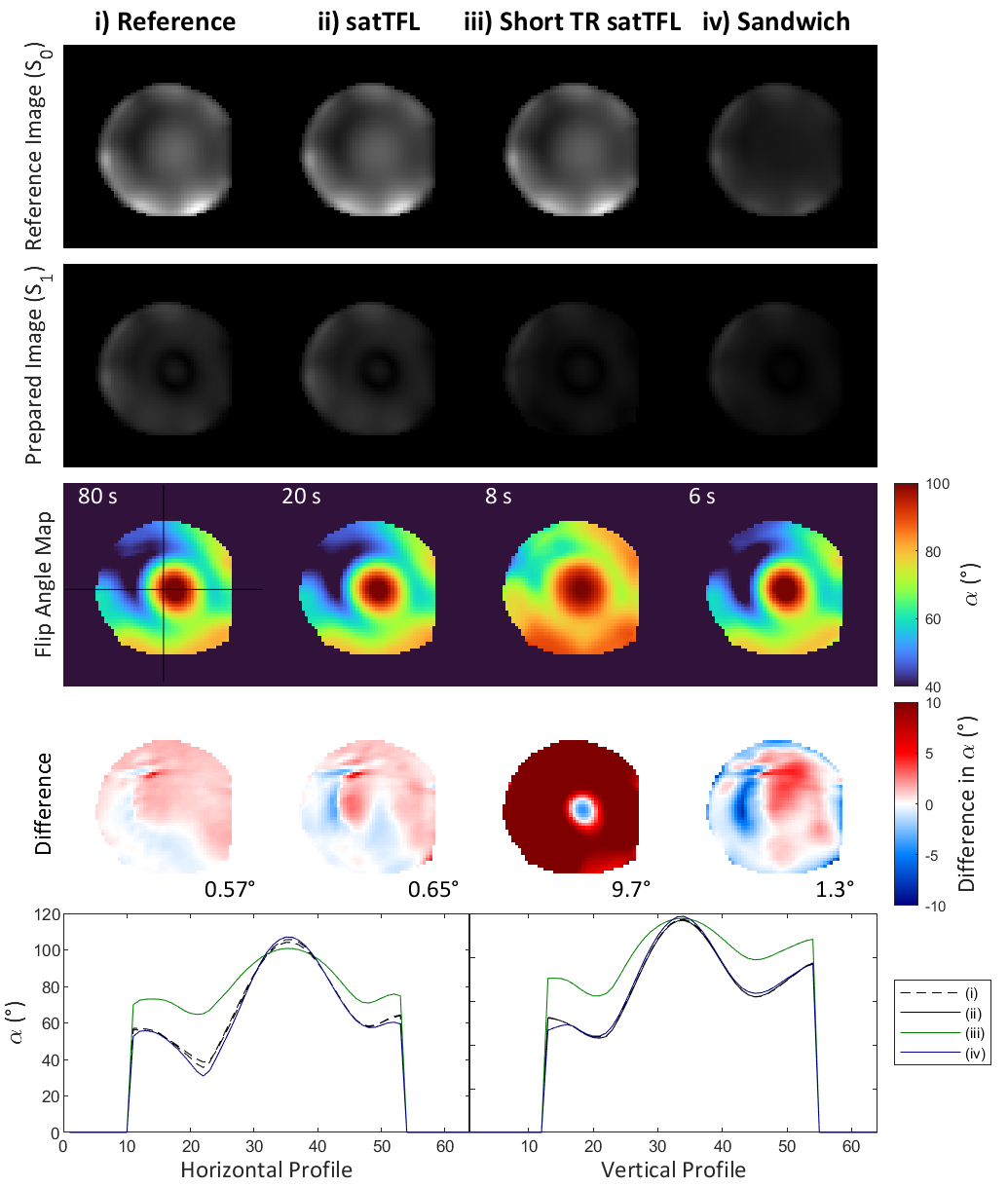
 *Figure S4: Flip angle maps from a water phantom at reference voltage 60 V for various sequences. i) segmented satTFL method with TR = 20 s ii) satTFL iii) satTFL acquired segmented and TR reduced to 2 s iv) segmented sandwiched scheme with TR = 1 s,* $\beta=0.1\alpha$*. The first two rows show the magnitude of the images used to calculate the corresponding flip angle maps in the third row. The difference between each of the maps at 60 V and a mean reference map, calculated from three reference voltages for the reference scheme and scaled by the voltage are shown in the fourth row, along with the RMSE. Flip angle profiles for horizontal and vertical profiles through the centre of the phantom are also shown. Note the reduced time to acquire iv) was 6 s compared to 80 s for i), 20 s for ii) and 8 s for iii). Note also that the dielectric properties of the water phantom give rise to much more inhomogeneous flip angle profiles than for the (non-polar) silicon oil phantom.*

*
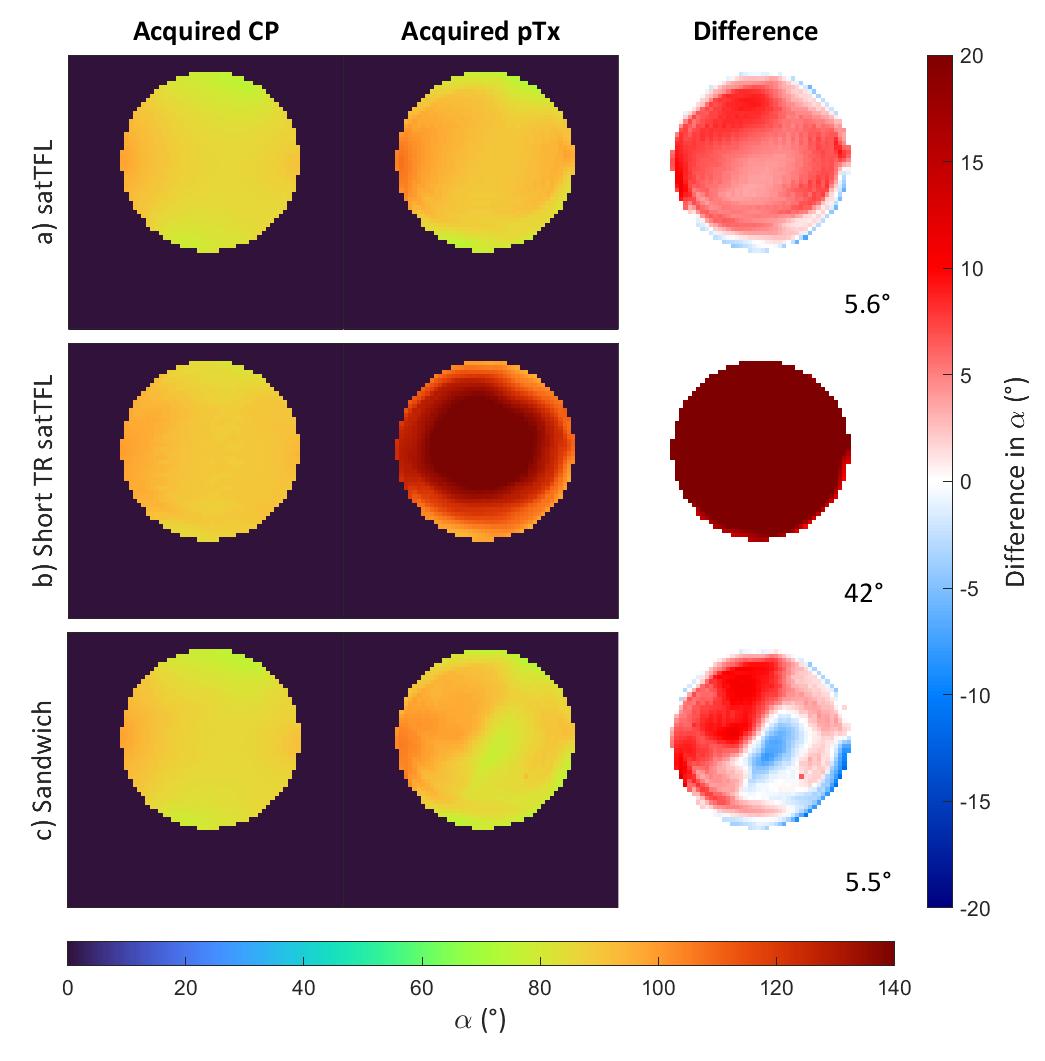

Figure S5: Comparison between acquired CP mode flip angle map and an (eight channel) pTx map for a silicon oil phantom.*

*
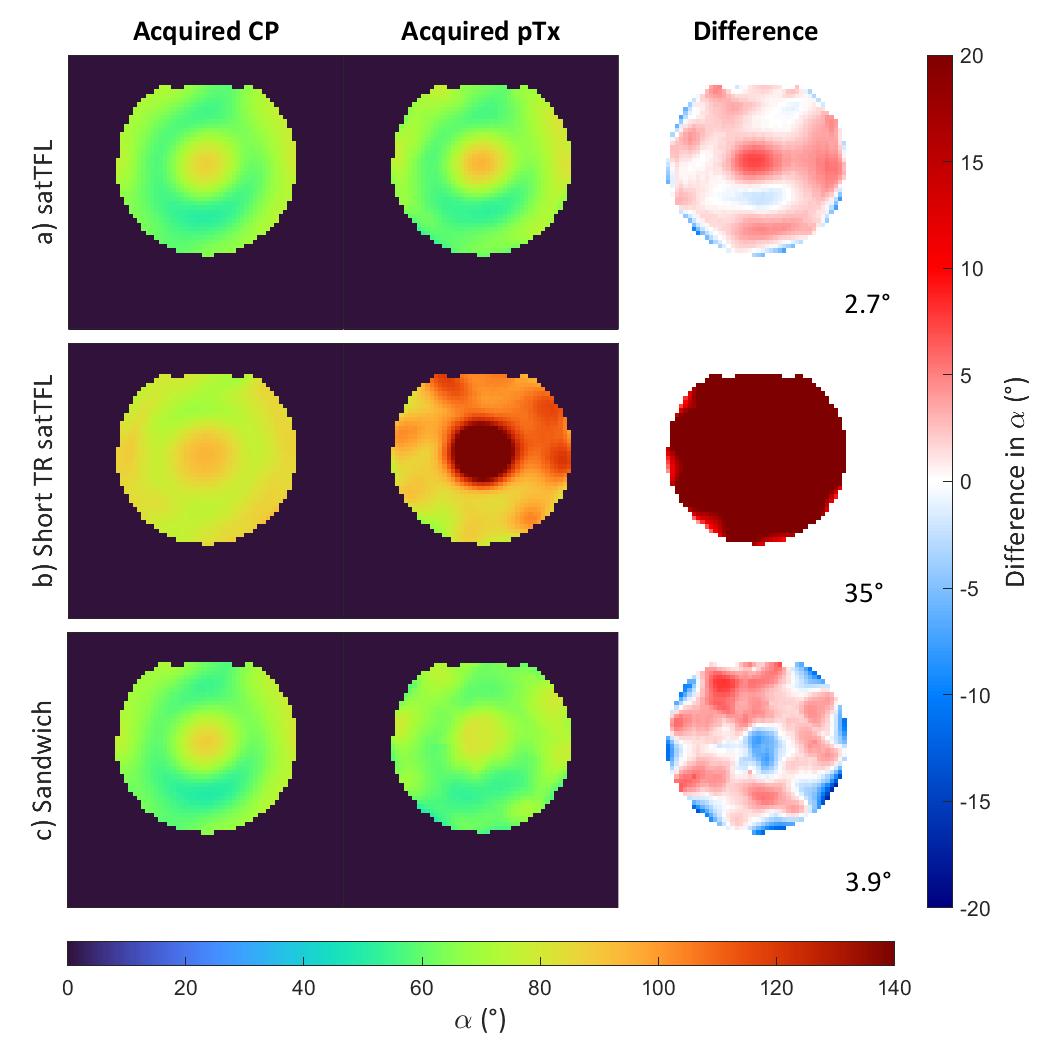

Figure S6: Comparison between acquired CP mode flip angle map and an (eight channel) pTx map for a water phantom.*

*
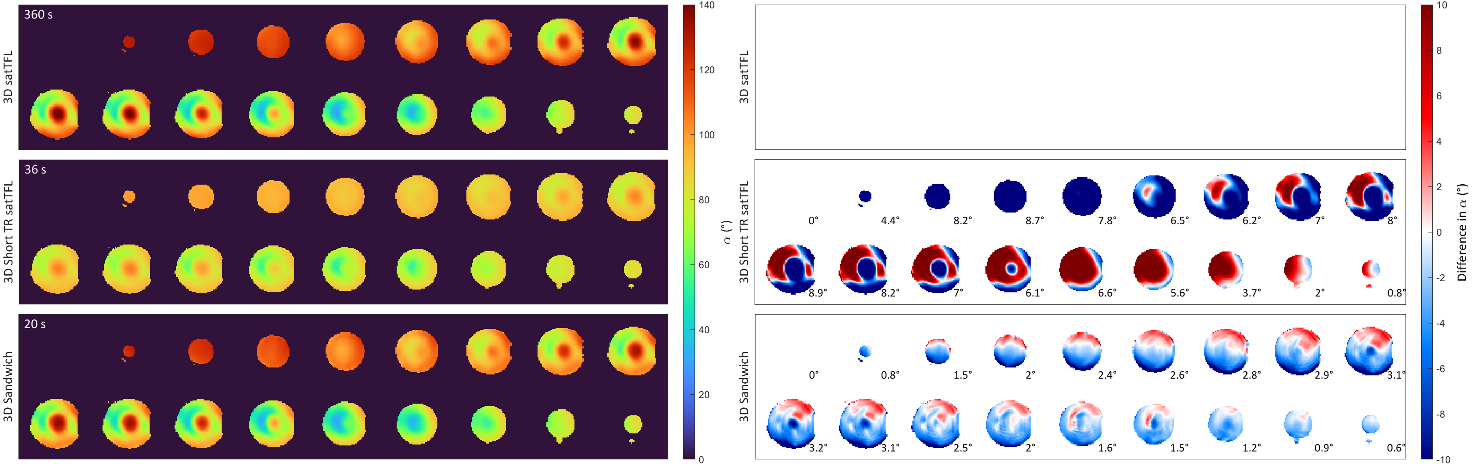
Figure S7: 3D B_1_^+^ maps for 18 partitions (10 mm) of the water phantom acquired using the 3D sequence in CP mode. On the left are maps acquired using satTFL, short TR satTFL and sandwich schemes for comparison. The difference and corresponding RMSE between satTFL and the other sequences is shown on the right. 3D data acquired in 20 s for our sandwich scheme and 36 s for short TR satTFL whereas this took 360 s with satTFL using a TR of 20 s.*

|  | 2D satTFL | 3D satTFL (Sinc $\boldsymbol{\alpha}$) | 3D satTFL  (HS8 $\boldsymbol{\alpha}$) | 3D Sandwich (Sinc $\boldsymbol{\alpha}$) | 3D Sandwich (HS8 $\boldsymbol{\alpha}$) | DREAM |
| --- | --- | --- | --- | --- | --- | --- |
| $\alpha$ type/ FA/ t_dur_ | Rectangular/ 90°/ 0.5 ms | Sinc/ 90°/ 5 ms | HS8/ 90°/ 5 ms | Sinc/ 90°/ 5 ms | HS8/ 90°/ 5 ms | Sinc/ 40°/ 0.7 ms |
| $\alpha$ pulse V_peak_ | 60 V | 180 V | 37 V | 180 V | 37 V | 80 V |
| $\beta$ type/ FA/ V_peak_/ t_dur_ | Rectangular/ 4°/ 14 V/ 0.1 ms | Rectangular/ 4°/ 14 V/ 0.1 ms | Rectangular/ 4°/ 14 V/ 0.1 ms | Rectangular/ 4°/ 14 V/ 0.1 ms | Rectangular/ 4°/ 14 V/ 0.1 ms | Sinc/ 15°/ 30 V/ 0.7 ms |
| TR | 20 s | 20 s | 20 s | 1 s | 1 s | 1 s |
| Matrix Size | 50×36×24 | 50×36×24 | 50×36×24 | 50×36×24 | 50×36×24 | 50×36×24 |
| Acquisition time | 480 s | 480 s | 480 s | 26 s (incl. 2 dummy TRs) | 26 s (incl. 2 dummy TRs) | 24 s |
| Average 10 s Power (Per Channel) | 0.001 W (S_0_) or  0.005 W (S_1_) | 0.001 W (S_0_) or  0.012 W (S_1_) | 0.001 W (S_0_) or  0.012 W (S_1_) | 0.135 W | 0.137 W | 0.141 W |
| Total Energy (Per Channel) | 1.54 J | 3.25 J | 3.28 J | 3.52 J | 3.56 J | 3.39 J |

*Table S1: Comparison of 10 s energy and total energy for satTFL, the sandwich scheme and DREAM using a reference voltage of 60 V. Power limits for the Nova head coil (8Tx/32Rx) were 12 W short-term (10 s average) and 24 W long-term (6 minute average). Sinc and HS8 preparation pulses have matched bandwidths of ±3 kHz. Sinc preparation pulses are likely hardware restricted by peak forward voltage.*
